# Association between metabolic syndrome and incidence of ocular motor nerve palsy

**DOI:** 10.1038/s41598-021-02517-3

**Published:** 2021-11-29

**Authors:** Daye Diana Choi, Kyungdo Han, Sei Yeul Oh, Kyung-Ah Park

**Affiliations:** 1 Department of Ophthalmology, Kim’s Eye Hospital, Seoul, South Korea; 2 Department of Statistics, Soong Sil University, Seoul, South Korea; 3 Department of Ophthalmology, Samsung Medical Center, Sungkyunkwan University School of Medicine, 81 Irwon-ro, Ilwon-dong, Gangnam-gu, Seoul, 06351 South Korea

**Keywords:** Medical research, Neurology

## Abstract

To assess the association between metabolic syndrome (MetS) and the development of third, fourth, and sixth cranial nerve palsy (CNP). Health checkup data of 4,067,842 individuals aged between 20 and 90 years provided by the National Health Insurance Service (NHIS) of South Korea between January 1, 2009, and December 31, 2009, were analyzed. Participants were followed up to December 31, 2017. Hazard ratio (HR) and 95% confidence interval (CI) of CNP were estimated using Cox proportional hazards regression analysis after adjusting for potential confounders. Model 1 included only incident CNP as a time-varying covariate. Model 2 included model 1 and individual’s age and sex. Model 3 included model 2, smoking status, alcohol consumption, and physical activity of individuals. We identified 5,835 incident CNP cases during the follow-up period (8.22 ± 0.94 years). Individuals with MetS (*n* = 851,004) showed an increased risk of CNP compared to individuals without MetS (*n* = 3,216,838) after adjustment (model 3: HR = 1.35, 95% CI 1.273–1.434). CNP incidence was positively correlated with the number of MetS components (log-rank *p* < 0.0001). The HR of CNP for males with MetS compared to males without MetS was higher than that of females with MetS compared to females without MetS (HR: 1.407, 95% CI 1.31–1.51 in men and HR: 1.259, 95% CI 1.13–1.40 in women, *p* for interaction = 0.0017). Our population-based large-scale cohort study suggests that MetS and its components might be risk factors for CNP development.

## Introduction

Metabolic syndrome (MetS) is a disorder characterized by a cluster of conditions. It can increase the risk of developing type 2 diabetes mellitus, cardiovascular disease, and all-cause mortality^1, 2^. Although suggested clinical criteria for the diagnosis of MetS vary somewhat according to the organization, main components of Mets include insulin resistance, hypertension, hypertriglyceridemia, low levels of high-density lipoprotein cholesterol (HDL-C), and obesity^3–5^. The prevalence of MetS has increased recently globally, including South Korea^6^. Data from the National Health and Nutrition Examination Survey (NHANES) III 1999–2006 showed that age-adjusted prevalence of MetS increased from 29.2% in 1999 to 34.2% in 2006 in the United States (US)^7^. According to data from the Korean National Health and Nutrition Examination Survey for 1998–2007, the prevalence of MetS in the general Korean population increased from 24.9% in 1998 to 31.3% in 2007^8^.

Third, fourth, and sixth cranial nerve palsies (CNP) are relatively common disorders. They can cause diplopia due to paralysis of the extraocular muscle. It is well-known that vasculopathic risk factors such as hypertension, diabetes, and dyslipidemia are predisposing factors for acquired ocular motor CNP^9–11^. However, to the best of our knowledge, studies on the relationship between ocular motor CNP and MetS, which represents a cluster of various conditions, have not been reported yet. Thus, the objective of the present study was to examine the association between MetS and third, fourth, and sixth CNP in Korean adult population using the National Health Insurance Service (NHIS)-National Sample Cohort (NSC) database.

## Methods

### Data source and study population

Our study used a database from the Korean National Health Insurance (NHI). Approximately 97% of the 50 million Korean population are covered by NHI while the remaining 3% of the population with low income are covered by the medical aid program. Subjects in the NHI database were identified by a unique Korean Resident Registration Number assigned to each South Korean resident at birth. This ensured no duplication or omission in the data. The medical provider must submit claims for reimbursement from the National Health Insurance Service (NHIS) for medical expenses. In this way, the NHIS gathers all healthcare utilization information including demographics, medical treatment, procedures, and disease diagnoses using codes from the International Classification of Diseases, 10th Revision-Clinical Modifications (ICD-10-CM)*.* In addition, the NHIS offers a national health screening program (NHSP) for all beneficiaries aged ≥ 20 years at least every 2 years. In the present study, we used a customized NHIS database cohort that included 40% of the Korean population who were selected by stratified random sampling to ensure that the sample was representative of the entire population. Among individuals aged between 20 and 90 years who participated in the NHSP in 2009, a total of 2,721,914 eligible individuals were finally identified after excluding those who had missing information (*n* = 43,312) and those who had a previous history of CNP or new CNP diagnosis or death within 1 year after the date of their health examination (*n* = 6802). We used this 1-year time lag in sensitivity analysis to avoid the problem of reverse causation. Therefore, eligible individuals were followed up for CNP cases from 1 year after the date of their health examination (the 1-year time lag) until December 31, 2017.

This study adhered to the tenets of the Declaration of Helsinki. It was approved by the Institutional Review Board of Samsung Medical Center (IRB; IRB no. SMC 2020-09-050). The IRB of Samsung Medical Center waived the requirement of informed consent from individual patients because data used were public and anonymized under confidentiality guidelines.

### Definition of ocular motor CNP and MetS

Ocular motor CNP was diagnosed based on ICD-10-CM code H49.0 (third CNP), H49.1 (fourth CNP), or H49.2 (sixth CNP), excluding anyone with comorbid dysthyroid exophthalmos (H05.2), thyrotoxicosis (E05), or myasthenia gravis (G70.0). MetS was defined based on the modified criteria of the National Cholesterol Education Program Adult Treatment Panel III with waist circumference (WC) cutoff modified for Asians^12, 13^. Individuals with at least three of the following components were diagnosed with MetS: (i) WC ≥ 90 centimeters (cm) for men or ≥ 85 cm for women; (ii) serum triglycerides ≥ 1.70 mmol/liter (mmol/L) or treatment with lipid-lowering medication; (iii) serum HDL-C < 1.04 mmol/L for men or < 1.30 mmol/L for women or treatment with lipid-lowering medication; (iv) systolic blood pressure (SBP) ≥ 130 mmHg, diastolic blood pressure (DBP) ≥ 85 mmHg, or treatment with antihypertensive medication; and (v) fasting plasma glucose ≥ 5.55 mmol/L or the use of hypoglycemic agents.

### Assessment

Standardized self-reported questionnaires were used to collect general health behavior and lifestyle information at the time of enrollment^14^ (Supplementary File 1). Smoking status was categorized into non-smokers, ex-smokers, and current smokers. Alcohol drinking was categorized into non-drinkers, mild to moderate drinkers, and heavy drinkers according to the amount of alcohol consumed on one occasion. Individuals who consumed over 30 g of alcohol per day were defined as heavy alcohol drinkers. Regular physical activity was defined as performing high-intensity exercise for at least 20 min three times per week or moderate-intensity exercise for at least 30 min five times per week. High‐intensity physical activity was defined as physical activity that caused extreme shortness of breath (e.g., running, bicycling at high speed, or carrying boxes upstairs). Moderate‐intensity physical activity was defined as physical activity that caused substantial shortness of breath (e.g., brisk walking, tennis, bicycling, carrying light boxes, and cleaning). Low-income level was defined as the lower quintile of the entire population.

Height (cm) and weight (kilogram [kg]) were measured using an electronic scale in medical institutions during health examinations. WC (cm) was measured at the middle point between the rib cage and iliac crest by trained examiners. Body mass index (BMI) was calculated as body weight in kg divided by height in meters squared (m^2^). General obesity was defined as a BMI of ≥ 25 kg/m^2^ based on the World Health Organization recommendations for Asian populations^15^. Abdominal obesity was defined as a WC of ≥ 90 cm for men and ≥ 85 cm for women according to the Asian-specific WC cutoff for abdominal obesity^16^. Blood samples were drawn after overnight fasting to measure serum levels of glucose, total cholesterol, triglycerides, HDL-C, low-density lipoprotein (LDL)-cholesterol, hemoglobin, serum creatinine, aspartate aminotransferase (AST), alanine aminotransferase (ALT), and gamma-glutamyl transpeptidase (γ-GTP).

Baseline comorbidities were identified based on past medical history with clinical and pharmacy codes of the ICD-10-CM. We defined hypertension as blood pressure (BP) of ≥ 140/90 mmHg or at least one claim per year for an antihypertensive medication prescription under ICD-10-CM codes I10-I13 and I15. Diabetes mellitus (DM) was defined by a fasting glucose of ≥ 126 mg/dL or at least one claim per year for a prescription of hypoglycemic agents under ICD-10-CM code E11-E14. Dyslipidemia was defined as a total cholesterol level of ≥ 240 mg/dL or at least one claim per year for a lipid-lowering medication prescription under ICD-10-CM code E78.

### Statistical analysis

Baseline characteristics of study participants according to the presence of MetS are presented as mean ± standard deviation for continuous variables and absolute frequencies for categorical variables. The Kolmogorov–Smirnov analysis to assess normal distribution of variables failed due to our large number of subjects. Instead, a histogram was drawn for each variable to confirm that it had a graph close to normal distribution (Supplementary File 2). Values were compared by independent t-test for continuous variables or chi-squared test for categorical variables. In Table 1, we analyzed the power and effect size for all inferential tests using the G*Power program. Incidence rates of ocular motor CNP were calculated by dividing the number of events by 1000 person-years. We performed multivariable Cox proportional hazards regression analysis to evaluate the association of MetS with incident ocular motor CNP and calculated the hazard ratios (HRs) and 95% confidence interval (CIs). Model 1 included only incident CNP as a time varying covariate. Model 2 included model 1 and individual’s age and sex. Model 3 included model 2, smoking status, alcohol consumption, and physical activity of individuals. In addition, we evaluated the risk of incident ocular motor CNP according to the coexistence of general obesity and MetS. We also conducted clinically relevant subgroup analyses and calculated p-values for interactions between MetS and subgroups in the development of ocular motor CNP using Cox regression analysis. All statistical analyses were carried out using SAS software version 9.4 (SAS Institute Inc., Cary, NC, USA).

**Table 1:** Baseline characteristics of the study population.

	Total		Male		Female	
	Metabolic syndrome		Metabolic syndrome		Metabolic syndrome	
	No	Yes	No	Yes	No	Yes
Number of patients, N (%)	3,216,838 (79.08)	851,004 (20.92)	1,720,737 (76.88)	517,336 (23.12)	1,496,101 (81.76)	333,668 (18.24)
Male, N (%)	1,720,737 (53.49)	517,336 (60.79)				
Age, mean ± SD	44.97 ± 13.63	54.97 ± 12.92	43.97 ± 13.36	51.24 ± 12.85	46.12 ± 13.84	60.76 ± 10.71
BMI, mean ± SD	23.09 ± 2.93	26.04 ± 3.23	23.53 ± 2.78	26.15 ± 3.1	22.6 ± 3.02	25.88 ± 3.42
WC, mean ± SD	78.24 ± 8.53	87.84 ± 8.95	81.65 ± 7.23	89.76 ± 8.35	74.33 ± 8.22	84.88 ± 9.04
Diabetes, N (%)	118,402 (3.68)	236,257 (27.76)	77,848 (4.52)	141,031 (27.26)	40,554 (2.71)	95,226 (28.54)
Hypertension, N (%)	538,516 (16.74)	556,754 (65.42)	300,523 (17.46)	313,975 (60.69)	237,993 (15.91)	242,779 (72.76)
Dyslipidemia, N (%)	301,629 (9.38)	437,893 (51.46)	149,688 (8.7)	222,327 (42.98)	151,941 (10.16)	215,566 (64.6)
Chronic kidney disease, N (%)	351,411 (10.92)	151,118 (17.76)	127,770 (7.43)	53,876 (10.41)	223,641 (14.95)	97,242 (29.14)
SBP, mean ± SD	119.81 ± 13.99	132.37 ± 14.7	122.13 ± 13.13	132.79 ± 14.08	117.15 ± 14.46	131.72 ± 15.58
DBP, mean ± SD	74.88 ± 9.52	81.73 ± 10.13	76.57 ± 9.12	82.75 ± 10	72.94 ± 9.6	80.16 ± 10.13
Total cholesterol, mean ± SD	192.29 ± 38.43	206.48 ± 48.53	191.82 ± 37.89	203.4 ± 47.09	192.84 ± 39.04	211.26 ± 50.31
HDL-C, mean ± SD	57.49 ± 31.73	52.8 ± 36.66	54.48 ± 29.57	50.79 ± 36.61	60.94 ± 33.71	55.93 ± 36.51
LDL-C, mean ± SD	122.01 ± 231.63	117.78 ± 122.02	120.55 ± 210.27	113.11 ± 137.96	123.69 ± 253.99	125.03 ± 91.53
Triglycerides, median (interquartile range)^a^	100.48 (100.43,100.54)	177.68 (177.48,177.89)	113.3 (113.21,113.39)	196.37 (196.08,196.66)	86.49 (86.42,86.56)	151.41 (151.14,151.68)
**Smoking**						
Never, N (%)	1,947,461 (60.54)	468,284 (55.03)	533,290 (30.99)	149,994 (28.99)	1,414,171 (94.52)	318,290 (95.39)
Former, N (%)	429,307 (13.35)	156,429 (18.38)	399,704 (23.23)	151,675 (29.32)	29,603 (1.98)	4754 (1.42)
Current, N (%)	840,070 (26.11)	226,291 (26.59)	787,743 (45.78)	215,667 (41.69)	52,327 (3.5)	10,624 (3.18)
**Drinking**						
Nondrinker, N (%)	1,644,759 (51.13)	449,134 (52.78)	561,867 (32.65)	161,631 (31.24)	1,082,892 (72.38)	287,503 (86.16)
Light to moderate drinker, N (%)	1,343,937 (41.78)	306,079 (35.97)	948,094 (55.1)	262,582 (50.76)	395,843 (26.46)	43,497 (13.04)
Heavy drinker, N (%)	228,142 (7.09)	95,791 (11.26)	210,776 (12.25)	93,123 (18)	17,366 (1.16)	2668 (0.8)
Regular PA, N (%)	570,179 (17.72)	167,920 (19.73)	341,959 (19.87)	110,340 (21.33)	228,220 (15.25)	57,580 (17.26)
Income, low, N (%)	565,511 (17.58)	144,028 (16.92)	249,557 (14.5)	78,890 (15.25)	315,954 (21.12)	65,138 (19.52)

*N* number, *SD* standard deviation, *BMI* body mass index, *WC* waist circumference, *SBP* systolic blood pressure, *DBP* diastolic blood pressure, *HDL-C* high-density lipoprotein cholesterol, *LDL-C* low-density lipoprotein cholesterol, *PA* physical activity.

^a^Geometric mean.

### Ethics approval

This study adhered to the tenets of the Declaration of Helsinki. It was approved by the Institutional Review Board of Samsung Medical Center (IRB; IRB no. SMC 2020-09-050). The requirement for informed consent from individual patients was waived because the data used were public and anonymized under confidentiality guidelines.

## Results

A total of 4,067,842 eligible participants were included in our cohort. At baseline, 851,004 individuals (20.92% of the total population) were diagnosed with MetS. Study participants were followed up until December 31, 2018, with an average follow-up duration of 8.22 ± 0.94 years. Table 1 shows baseline characteristics of the study population according to the presence of MetS. The proportion of men was higher in the MetS group than in the non-MetS group (60.79% vs. 53.49%). The mean age was 54.97 ± 12.92 years in the MetS group and 44.97 ± 13.63 years in the non-MetS group. The mean age was 51.24 ± 12.85 years for men and 60.76 ± 10.71 years for women in the MetS group. Individuals with MetS more frequently had DM, hypertension, dyslipidemia, and chronic kidney disease. The MetS group also exhibited higher mean BMI, WC, DBP, SBP, serum total cholesterol, and triglyceride values than the non-MetS group. Mean LDL-C and HDL-C values were lower in individuals with MetS than in those without MetS. Proportions of ex-smokers, current smokers, and heavy drinkers were higher in the MetS group than in the non-MetS group. Proportions of people with regular exercise and low income were higher in the MetS group than in the non-MetS group. All variables were significant (*p*-values < 0.001). This appeared to result from the very large sample size. We also calculated the power and effect size (absolute standardized difference, ASD) for each variable in Table 1. The power was 1 for all statistical tests in Table 1 due to the large sample size.

A total of 5835 individuals were diagnosed with ocular motor CNP during the follow-up period. The incidence rate of ocular motor CNP in the MetS group was approximately 2.19 times higher than that in the non-MetS group (Table 2). Compared to the non-MetS group, the MetS group showed an increased risk of ocular motor CNP development in all models (HR, 95% CI: model 1 = 2.19, 2.08–2.31; model 2 = 1.43, 1.35–1.51; and model 3 = 1.35, 1.27–1.43). Each component of MetS showed a similar association with the incidence of ocular motor CNP even after adjusting for confounding variables. The presence of abdominal obesity had an approximately 24% higher risk of developing ocular motor CNP compared to no abdominal obesity (model 3, HR = 1.24, 95% CI 1.17–1.31). Individuals with high fasting plasma glucose levels had an approximately 43% higher risk of ocular motor CNP compared to those without (model 3, HR = 1.43, 95% CI 1.35–1.51). Individuals with hypertriglyceridemia had an HR of 1.18 (95% CI 1.12–1.25). Those with low HDL-C had an HR of 1.24 (95% CI 1.16–1.33) for ocular motor CNP in model 3. High BP was also significantly associated with an increased risk of ocular motor CNP (model 3, HR, 95% CI 1.13, 1.06–1.19).

**Table 2:** Risk of ocular motor cranial nerve palsy according to the presence of metabolic syndrome and its components.

	N	Event	Person-years	Incidence rate^a^	HR (95% CI)		
					Model 1^b^	Model 2^c^	Model 3^d^
**MetS**							
No	3,216,838	3713	26,508,066.95	0.14007	1 (Ref.)	1 (Ref.)	1 (Ref.)
Yes	851,004	2122	6,910,285.61	0.30708	2.193 (2.079,2.313)	1.427 (1.35,1.507)	1.351 (1.273,1.434)
**WC (cm)**							
M < 90, F < 85	3,269,478	4175	26,894,123.84	0.15524	1 (Ref.)	1 (Ref.)	1 (Ref.)
M ≥ 90, F ≥ 85	798,364	1660	6,524,228.72	0.25444	1.639 (1.548,1.735)	1.237 (1.167,1.31)	1.239 (1.17,1.313)
**Serum triglycerides (mmol/L)**							
Low (< 1.70)	2,639,483	3106	21,717,870.01	0.14302	1 (Ref.)	1 (Ref.)	1 (Ref.)
High (≥ 1.70)	1,428,359	2729	11,700,482.55	0.23324	1.631 (1.549,1.717)	1.245 (1.182,1.311)	1.183 (1.121,1.248)
**Serum HDL-C (mmol/L)**							
High (M ≥ 1.04, F ≥ 1.30)	3,630,818	4754	29,857,072.14	0.15923	1 (Ref.)	1 (Ref.)	1 (Ref.)
Low (M < 1.04, F < 1.30)	437,024	1081	3,561,280.41	0.30354	1.905 (1.783,2.035)	1.296 (1.211,1.386)	1.242 (1.16,1.329)
**BP**							
Normal	2,262,740	2305	18,733,463.27	0.12304	1 (Ref.)	1 (Ref.)	1 (Ref.)
High^e^	1,805,102	3530	14,684,889.28	0.24038	1.955 (1.855,2.06)	1.179 (1.115,1.247)	1.126 (1.063,1.192)
**Plasma fasting glucose**							
Normal	2,791,821	3038	23,050,149.11	0.1318	1 (Ref.)	1 (Ref.)	1 (Ref.)
High^f^	1,276,021	2797	10,368,203.44	0.26977	2.049 (1.946,2.157)	1.462 (1.387,1.541)	1.428 (1.354,1.506)
**Smoking**							
Non	2,415,745	3177	19,904,128.18	0.15962	1 (Ref.)	1 (Ref.)	1 (Ref.)
Ex	585,736	1159	4,790,690.26	0.24193	1.519 (1.42,1.625)	1.070 (0.99,1.156)	1.094 (1.011,1.183)
Current	1,066,361	1499	8,723,534.12	0.17183	1.079 (1.015,1.147)	1.051 (0.977,1.131)	1.104 (1.024,1.19)
**Drinking**							
Non	2,093,893	3289	17,156,972.09	0.1917	1 (Ref.)	1 (Ref.)	1 (Ref.)
Mild	1,650,016	2079	13,612,474.28	0.15273	0.797 (0.755,0.842)	0.887 (0.835,0.943)	0.872 (0.82,0.928)
Heavy	323,933	467	2,648,906.18	0.1763	0.921 (0.836,1.015)	0.865 (0.782,0.958)	0.832 (0.75,0.923)
**Physical activity**							
No	3,329,743	4623	27,336,646.48	0.16911	1 (Ref.)	1 (Ref.)	1 (Ref.)
Yes	738,099	1212	6,081,706.08	0.19929	1.178 (1.105,1.254)	1.015 (0.952,1.081)	1.013 (0.95,1.079)

*N* number, *HR* hazard ratio, *CI* confidence interval, *MetS* metabolic syndrome, *WC* waist circumference, *M* males, *F* females, *HDL-C* high-density lipoprotein cholesterol, *BP* blood pressur.

^a^Ocular motor incidence per 1,000 person-years.

^b^Model 1 was unadjusted.

^c^Model 2 was adjusted for age and sex.

^d^Model 3 was adjusted for age, sex, smoking status, alcohol consumption, and physical activity.

^e^Systolic BP ≥ 130 mmHg, diastolic BP ≥ 85 mmHg, or treatment with antihypertensive agents.

^f^Plasma fasting glucose ≥ 5.55 mmol/L or the use of hypoglycemic agents.

**Figure 1:**
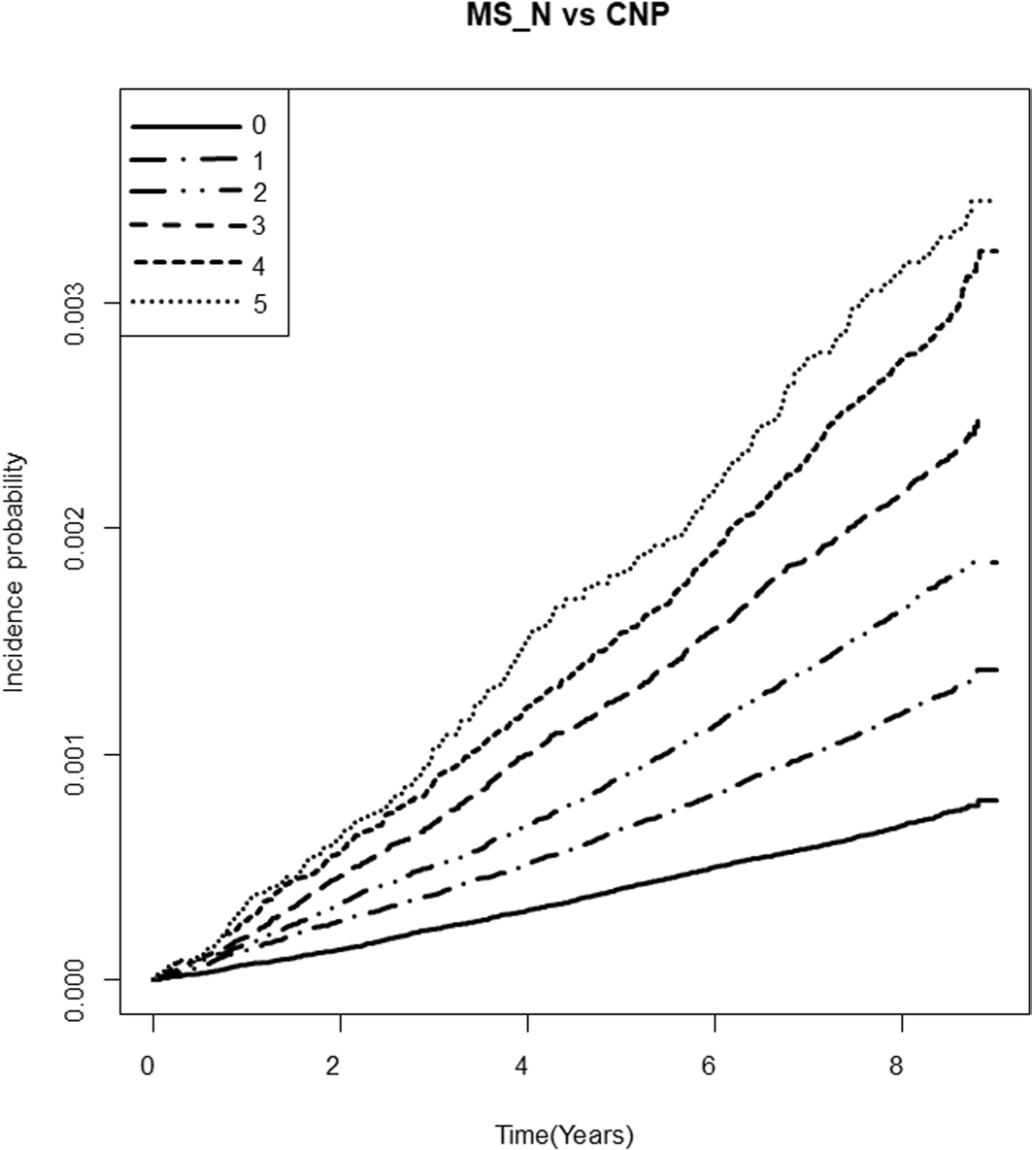
Kaplan–Meier curves of incidence probability of ocular motor cranial nerve palsy (CNP) up to 8 years according to the number of metabolic syndrome (MetS) components. Having more MetS components was associated with an increased risk of developing ocular motor CNP during the follow-up period compared to having no MetS component (log-rank test *p* > 0.0001).

The Kaplan Meier curve in Fig. 1 and Table 3 presents the incidence probability of ocular motor CNP according to the number of MetS components compared to the group without any components. Ocular motor CNP incidence was positively correlated with the number of MetS components (log-rank *p* < 0.0001). The HR for incident ocular motor CNP compared to people without any MetS components gradually increased with the number of components (*p* for trend < 0.0001) (Table 3). These associations persisted even after adjusting for potential confounding variables including smoking status, alcohol consumption, and physical activity. Individuals with three MetS components were at 65% higher risk of developing ocular motor CNP (model 3, HR, 95% CI 1.65, 1.51–1.81). Those with all five components were at a 97% higher risk of developing ocular motor CNP compared to those without any components (model 3, HR, 95% CI 1.97, 1.70–2.26).

**Table 3:** Incidence probability of ocular motor cranial nerve palsy up to 8 years according to the number of metabolic syndrome components.

N of MetS components	N	Event	Incidence rate^a^	Model 1^b^	Model 2^c^	Model 3^d^
0	1,264,973	912	0.087	1 (ref.)	1 (ref.)	1 (ref.)
1	1,130,746	1403	0.151	1.736 (1.597,1.887)	1.209 (1.111,1.396)	1.214 (1.116,1.322)
2	821,119	1398	0.208	2.4 (2.207,2.608)	1.384 (1.27,1.508)	1.396 (1.281,1.522)
3	511,220	1139	0.274	3.152 (2.889,3.439)	1.632 (1.491,1.786)	1.65 (1.507,1.807)
4	260,694	734	0.348	4.004 (3.633,4.413)	1.874 (1.693,2.073)	1.894 (1.711,2.096)
5	79,090	249	0.390	4.498 (3.91,5.175)	1.948 (1.687,2.249)	1.965 (1.702,2.269)
*p* for trend				< 0.0001	< 0.0001	< 0.0001

*N* number, *MetS* metabolic syndrome.

^a^Ocular motor cranial nerve palsy incidence per 1,000 person-years.

^b^Model 1 was unadjusted.

^c^Model 2 was adjusted for age and sex.

^d^Model 3 was adjusted for age, sex, smoking status, alcohol consumption, and physical activity.

There was a significant interaction between sex and MetS on the risk of ocular motor CNP (*p* for interaction = 0.0017). The HR for males with MetS to develop ocular motor CNP compared to males without MetS was higher than that for females with MetS compared to females without MetS (HR, 95% CI 1.407, 1.31–1.51 in men and 1.259, 1.13–1.40 in women).

## Discussion

Criteria for the diagnosis of MetS have been suggested by various organizations including the World Health Organization (WHO), the European Group for the Study of Insulin Resistance (EGIR), the National Cholesterol Education Program—Third Adult Treatment Panel (NCEP ATP III), the American Association of Clinical Endocrinologists (AACE), and the International Diabetes Federation (IDF)^3–5, 12, 13, 15^. All groups agree on critical components of metabolic syndrome: obesity, insulin resistance, dyslipidemia, and hypertension. This study used the NCEP ATP III criteria for hypertension, hypertriglyceridemia, low HDL-C, and hyperglycemia. For abdominal obesity, this study used the definition in Korean clinical practice guidelines. MetS affects nearly 30% of the world’s population and causes a two to threefold increase in morbidity and mortality compared to healthy people^17^. In Korea, a 22.4–32.1% prevalence of MetS has been reported^8, 18, 19^. MetS has been associated not only with cardiovascular disease and type 2 DM, but also with diverse diseases such as cancer^20, 21^, respiratory disease^22^, and chronic kidney disease^23^. The association between MetS and neurodegenerative diseases such as Alzheimer’s disease and multiple sclerosis has also gained attention^24, 25^.

Our population-based cohort study found that the incidence of ocular motor CNP was 2.19 times greater for individuals with MetS compared to those without. Individuals with MetS had a 35% higher risk of incident ocular motor CNP than individuals without MetS during the mean follow-up period of 8 years. Each component of MetS was significantly associated with a higher risk of incident ocular motor CNP. As the number of MetS components increased, the risk of incident ocular motor CNP gradually increased. The hazard ratio for ocular motor CNP in males with MetS compared to males without MetS was higher than that in females with MetS compared to females without MetS.

In our study, a high fasting plasma glucose and an abdominal obesity were associated with a 43% increase and a 24% increase, respectively, in the risk of developing ocular motor CNP after adjusting possible confounding factors. Hyperglycemia affects the tricarboxylic acid (TCA) cycle and glycation reactions, resulting in oxidative stress^26^. Furthermore, advanced glycation end products (AGEs) can promote the inflammation cascade by activating AGE receptors on immune cells^27^. Based on these concepts, diabetes can be thought of as a chronic inflammatory disease^28^. Oxidative stress and inflammation, especially the overproduction of reactive oxygen species (ROS) from partial reduction of O_2_, can cause mitochondrial dysfunction in many cells including neurons. Due to high metabolic activity and dependence on energy supply, mitochondrial damage from oxidative stress such as ROS can cause nerve cell damage^29^. Moreover, leptin, a signaling molecule produced by adipocytes that acts on the hypothalamus to increase satiety, is overproduced in obese individuals. High plasma concentrations of leptin can induce leptin resistance in the brain. Thus, obese people continue to feel hungry. Leptin is also involved in immune modulation, such as leukocyte extravasation and the development and activation of leukocytes^30^. These pro-inflammatory environments in both obesity and type 2 diabetes mellitus contribute to the increased permeability of the blood–brain-barrier (BBB)^31^. BBB breakdown causes decreased removal of waste and increased infiltration of immune cells, leading to disruption of glial and neuronal cells.

In one previous study, diabetes and WC were the main metabolic factors associated with polyneuropathy, whereas SBP, triglyceride levels, and HDL-C levels were not^32^. In our study, not only diabetes, but also hypertension, high triglyceride levels, and low HDL-C levels significantly increased the risk of ocular motor CNP. The prevalent coexistence of hypertension^10, 33^, obesity^33^, and dyslipidemia^11^ has been reported in ocular motor CNP patients. In one clinical study on the etiology of ocular motor CNP, hypertension and dyslipidemia along with DM were prevalent not only in patients with presumed microvascular ischemia but also in patients with other identifiable causes^11^. In our study, the presence of hypertension, elevated serum levels of triglycerides, and decreased levels of HDL-C increased the risk of ocular motor CNP by 13%, 18%, and 24%, respectively.

Individuals with a higher number of MetS components were at a higher risk of incident ocular motor CNP in this study. Our results suggest that each component of MetS has an additive effect on the risk of the development of ocular motor CNP. We presume that treating MetS as a whole might be of value to reduce the incidence of ocular motor CNP. However, due to limitations of a correlation study, we were unable to confirm the causative effect of MetS on ocular motor CNP. Further studies are warranted to explore the effect of MetS treatment on the development and progression of ocular motor CNP.

The HR of ocular motor CNP for males with MetS compared to males without MetS was higher than that for females with MetS compared to females without MetS. This finding suggests that men with MetS are more vulnerable to ocular motor CNP than women with MetS. Also, the proportion of males (23.12%) in the MetS group was higher than that of females (18.24%) and the mean age of the males in the MetS group was lower than that of the females in our cohort. These results suggested that men in our cohort were more prone to MetS and that men with MetS were more prone to ocular motor CNP. Although the underlying mechanism is currently unclear, sex differences in body fat distribution, glucose homeostasis, and lipid metabolism have been previously reported^34, 35^. Women generally have higher adiposity relative to men throughout their entire lifespan. However, men often have higher adipose tissue distributed in the central or abdominal subcutaneous region. This distribution might be predominantly sex hormone-dependent^36^. Subcutaneous abdominal fat has been correlated with an increased susceptibility for MetS. Visceral lower body fat has been associated with reduced metabolic risk. It might be protective against adverse effects of obesity. Furthermore, visceral adipocytes in men exhibit higher rates of fatty acid turnover and lipolysis than those in women, leading to a greater release of free fatty acid into the circulation. Both sex hormones and sex chromosome complement may play roles in such differences. However, relatively few studies have focused on the underlying mechanisms^37, 38^. Gender may not only affect obesity, but also affect neurological complications. In an experimental study using diabetic mice, male mice developed greater diabetes-induced cognitive deficits and peripheral neurovascular dysfunction than female mice^39^. A human study comparing male and female diabetic patients showed that males developed neuropathic complications 4 years earlier than females^40^.

This study has several limitations. They should be considered when interpreting our results. First, this was a retrospective cohort case–control study. Thus, our data could only suggest the correlation between MetS and ocular motor CNP. The causative relationship between the two could not be predicted. Second, the status of MetS changes over time. However, we used single, fixed measures at baseline. Thus, in this study, we do not know whether an improvement in MetS would decrease the risk of ocular motor CNP development. Third, since our study used insurance data comprised of diagnostic codes, there might be a possibility of misdiagnosis. Moreover, due to the lack of data, some possible confounding factors such as dietary factors that may affect Mets or the duration of Mets could not be evaluated. Lastly, our data were comprised of mostly Koreans. Thus, our results might not be applicable to other ethnicities. However, the present study was a population-based, nationwide large-scale cohort study with merits to offset its limitations. Also, our study suggested the harmful effect of MetS on the development of ocular motor CNP in the general population for the first time to the best of our knowledge.

In conclusion, we found that MetS and its components were independent risk factors for ocular motor CNP development using a population-based sample over 8 years. Components of MetS had additive effects. An increased number of MetS components was associated with an increased risk of ocular motor CNP incidence, suggesting the importance of an integrated risk management approach for MetS. Males with MetS vs. males without MetS had a higher hazard ratio of experiencing ocular motor CNP than females with MetS vs. females without MetS.

## Supplementary Information

Supplementary Information 1.

Supplementary Information 2.

## Data Availability

The data that support the findings of this study are available from Korean National Health Insurance (NHI). However, restrictions apply to the availability of these data, which were used under permission for the current study as such data are not publicly available. Data are however available upon reasonable request and with permission of Korean National Health Insurance (NHI).
