# Supplementary material for: Association between metabolic syndrome and incidence of ocular motor nerve palsy
Source: Sci Rep. 2021 Nov 29;11:23033. doi: 10.1038/s41598-021-02517-3 (PMC8630222; doi:10.1038/s41598-021-02517-3)
Supplement: Supplementary file 1 — Supplementary Information 1. [file 41598_2021_2517_MOESM1_ESM.pdf]

Health checkup questionnaire

※ Examinees must complete the questionnaire to receive the results of the cardiovascular disease risk assessment.

Last Name		Resident Reg. No..		Telephone	Home	
Given Name					Mobile phone	
Current address				E-mail		
				How to receive a health checkup report		□ Post □ E-mail

※ Please answer all the questions below.

Medical history (disease history, family history)

1. Have you ever been diagnosed by a doctor with any of the following diseases or are you currently taking any medication?

	Diagnosis		Medication therapy	
Brain stroke (paralysis)	Yes	No	Yes	No
Cardiac infarction/angina	Yes	No	Yes	No
High blood pressure	Yes	No	Yes	No
Diabetes	Yes	No	Yes	No
Dyslipidemia	Yes	No	Yes	No
Tuberculosis	Yes	No	Yes	No
Others (including cancer)	Yes	No	Yes	No

2. Has anyone in your family died from or gotten any of the following diseases?

Brain stroke (paralysis)	Yes	No
Cardiac infarction/angina	Yes	No
High blood pressure	Yes	No
Diabetes	Yes	No
Others (including cancer)	Yes	No

3. Are you a Hepatitis B virus antigen carrier?
① Yes ② No ③ No idea

Smoking and e-cigarettes (vaping)

4. Have you ever smoked more than 5 packs of cigarettes (100 cigarettes) in your lifetime?
① No. (☞ Go to Question 5)
② Yes. (☞ Go to Question 4-1)

4-1. Do you smoke cigarettes now?

① I do	A total of ____ years	An average of _______ cigarettes a day	
② I used to but not anymore	A total of ____ years	Used to smoke _______ cigarettes a day on average	____ years since I quitted

5. Have you ever smoked an electronic cigarette (e.g., IQOS, Glo, or Lil)?
① No. (☞ Go to Question 6)
② Yes. (☞ Go to Question 5-1)

5-1. Do you smoke electronic cigarettes now?

① I do	A total of ____ years	An average of _______ cigarettes a day	
② I used to but not anymore	A total of ____ years	Used to smoke _______ cigarettes a day on average	____ years since I quitted

6. Have you ever used a liquid electronic cigarette?
① No.
② Yes. (☞ Go to Question 6-1)

6-1. Have you used a liquid electronic cigarette in the last month?
① No ② 1 to 2 days per month ③ 3 to 9 days per month
④ 10 to 29 days per month ⑤ Every day

Drinking

※ In the past one year

7. How often do you have drinks containing alcohol? (Select one)
① (   ) times per week ② (   ) times per month
③ (   ) times per year
④ I don't drink alcohol.

7-1. How many drinks containing alcohol do you have on a typical day when you are drinking?
* <u>Choose one among the glass, bottle, can, or cc (you can choose more than one for liquor types; choose a similar type for other liquor types that are not indicated)</u>

Type of liquor	Glass	Bottle	Can	cc
Soju				
Beer				
Hard liquor				
Makgeolli (rice wine)				
Wine				

7-2. What is the largest amount of drinks containing alcohol that you have ever had in one day?
* <u>Choose one among the glass, bottle, can, or cc (you can choose more than one for liquor types, choose a similar type for other liquor types that are not indicated)</u>

Type of liquor	Glass	Bottle	Can	cc
Soju				
Beer				
Hard liquor				
Makgeolli (rice wine)				
Wine				

Exercising

8-1. How often do you do high intensity exercise (making you short of breath) per week?
(   ) days per week
* Examples of high intensity exercise> Running, aerobics, fast bicycling, construction labor, carrying items using stairs, etc.

8-2. How long do you do high intensity exercise (making you short of breath) per day?
(   ) hours (   ) minutes per day

9-1. How often do you do moderate intensity exercise (making you slightly short of breath) per week?
(   ) days per week
* Exclude exercise you have already written in Question 8
* Examples of moderate intensity exercise> Power walking, doubles tennis games, cycling at normal speed, carrying light items, cleaning, etc.

9-2. How long do you do moderate intensity exercise (making you slightly short of breath) per day?
(   ) hours (   ) minutes per day

10. How many days did you do weight training such as push-ups, sit-ups, dumbbell exercises, weight lifting, or horizontal bar exercise in the last one week?
(   ) days per week
