# Supplementary figures and images for: Association between metabolic syndrome and incidence of ocular motor nerve palsy
Source: Sci Rep. 2021 Nov 29;11:23033. doi: 10.1038/s41598-021-02517-3 (PMC8630222; doi:10.1038/s41598-021-02517-3)

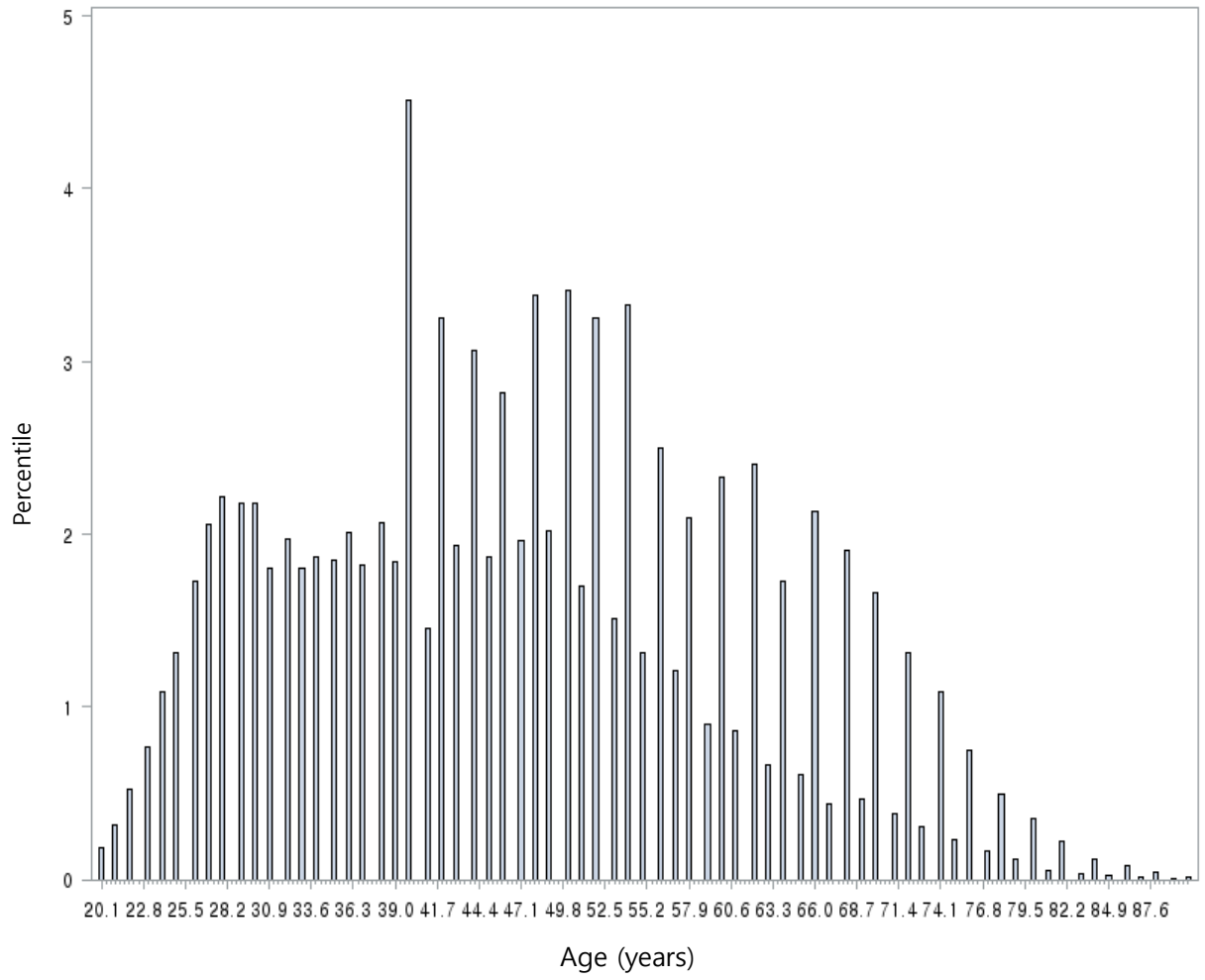

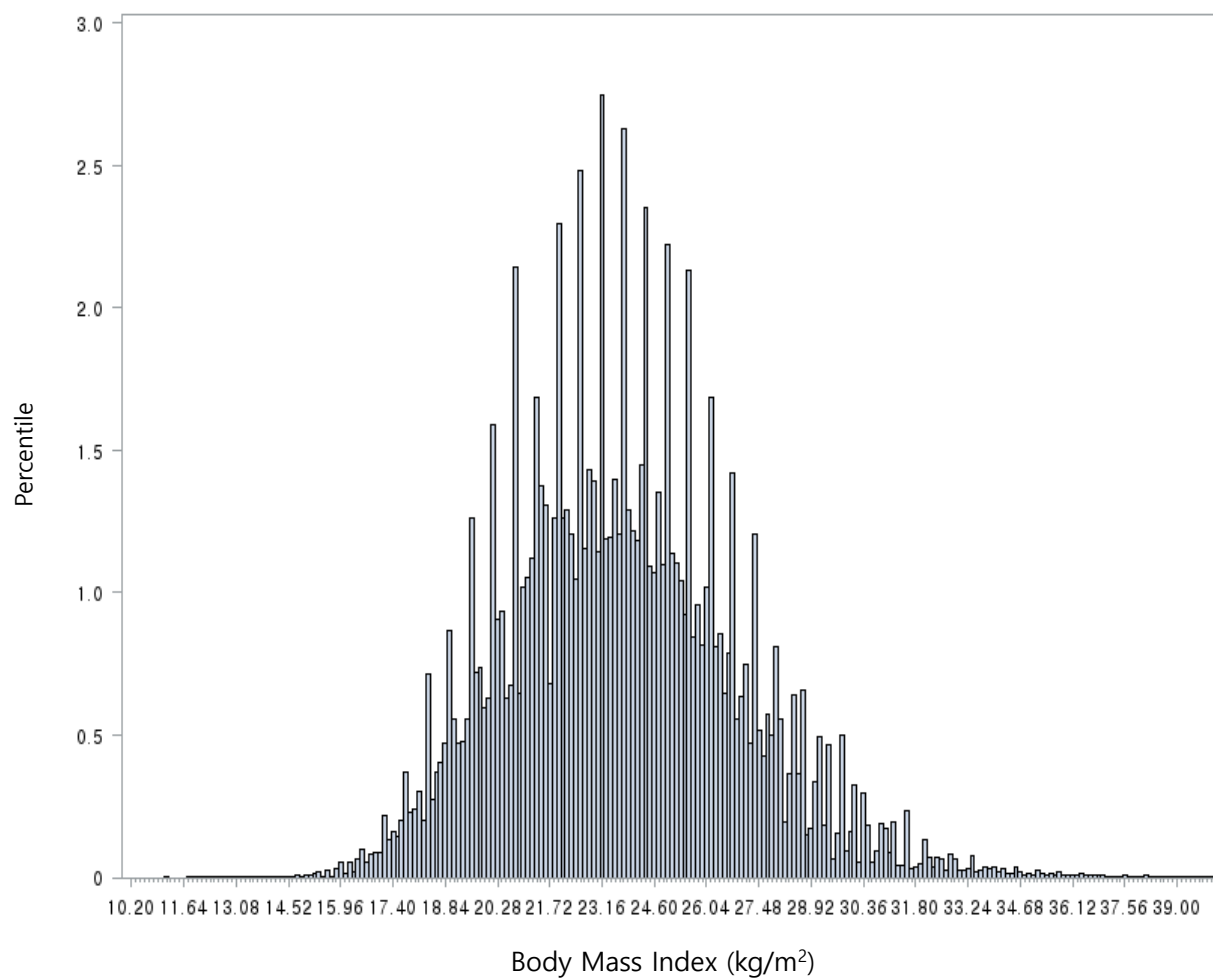

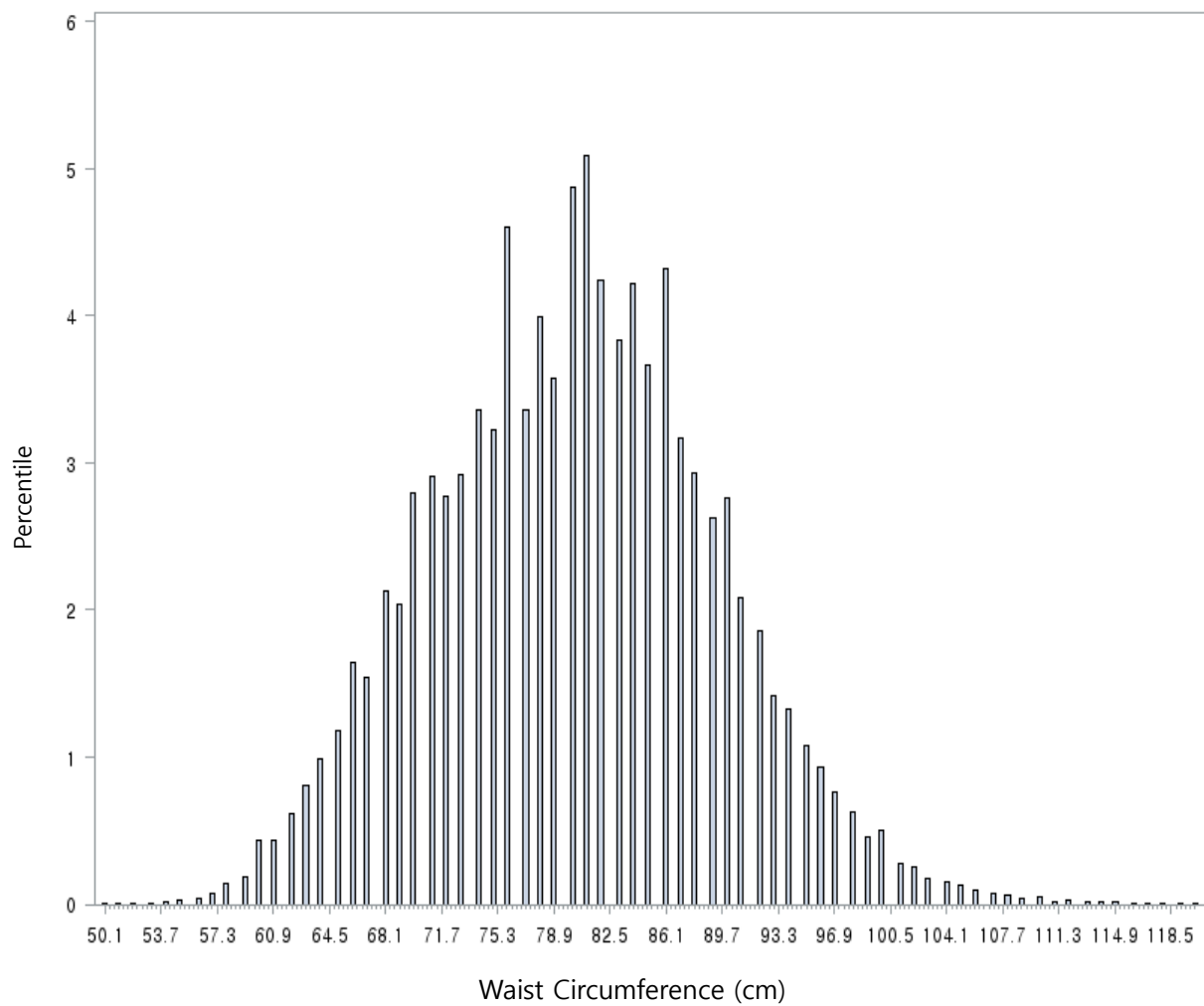

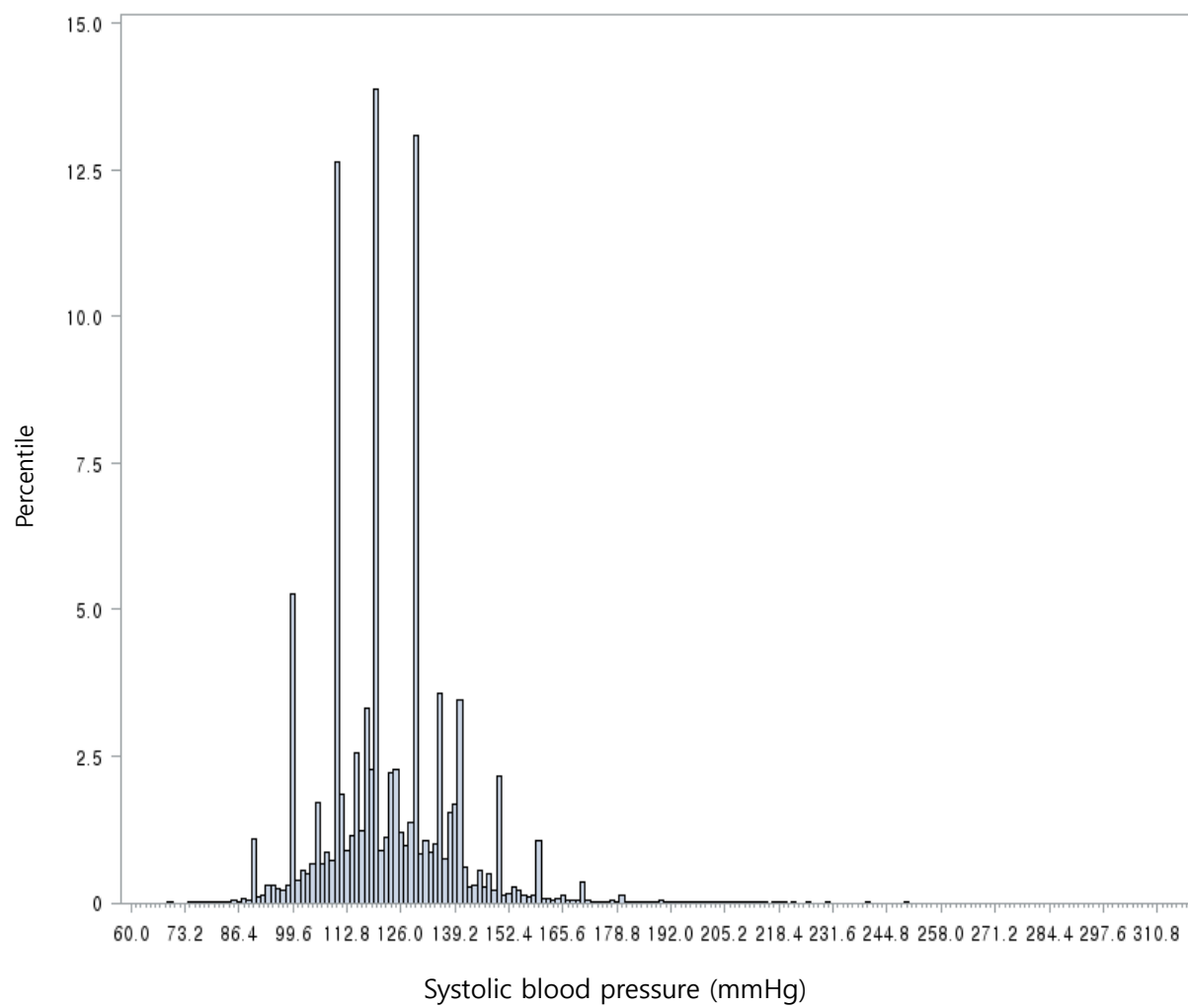

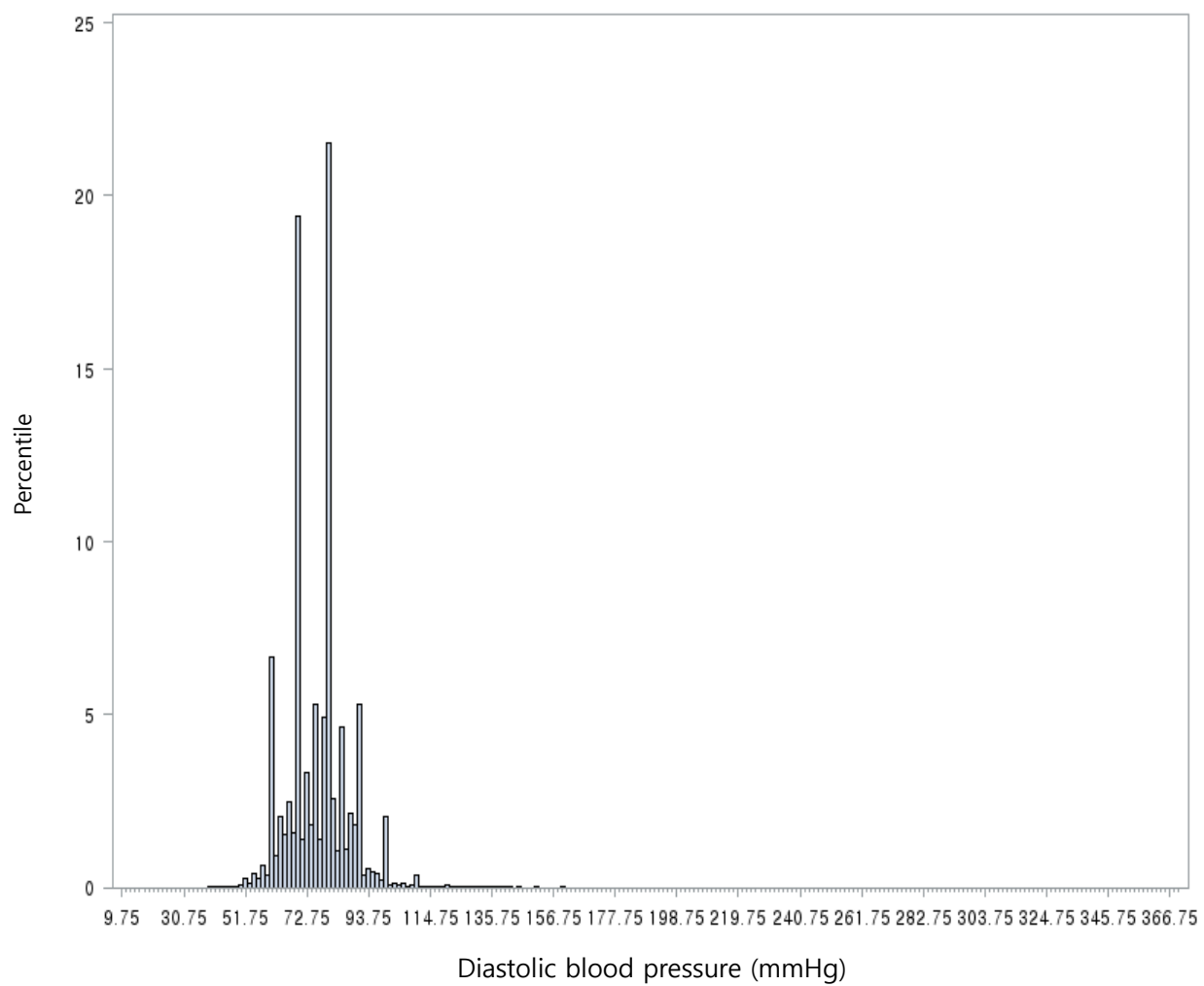

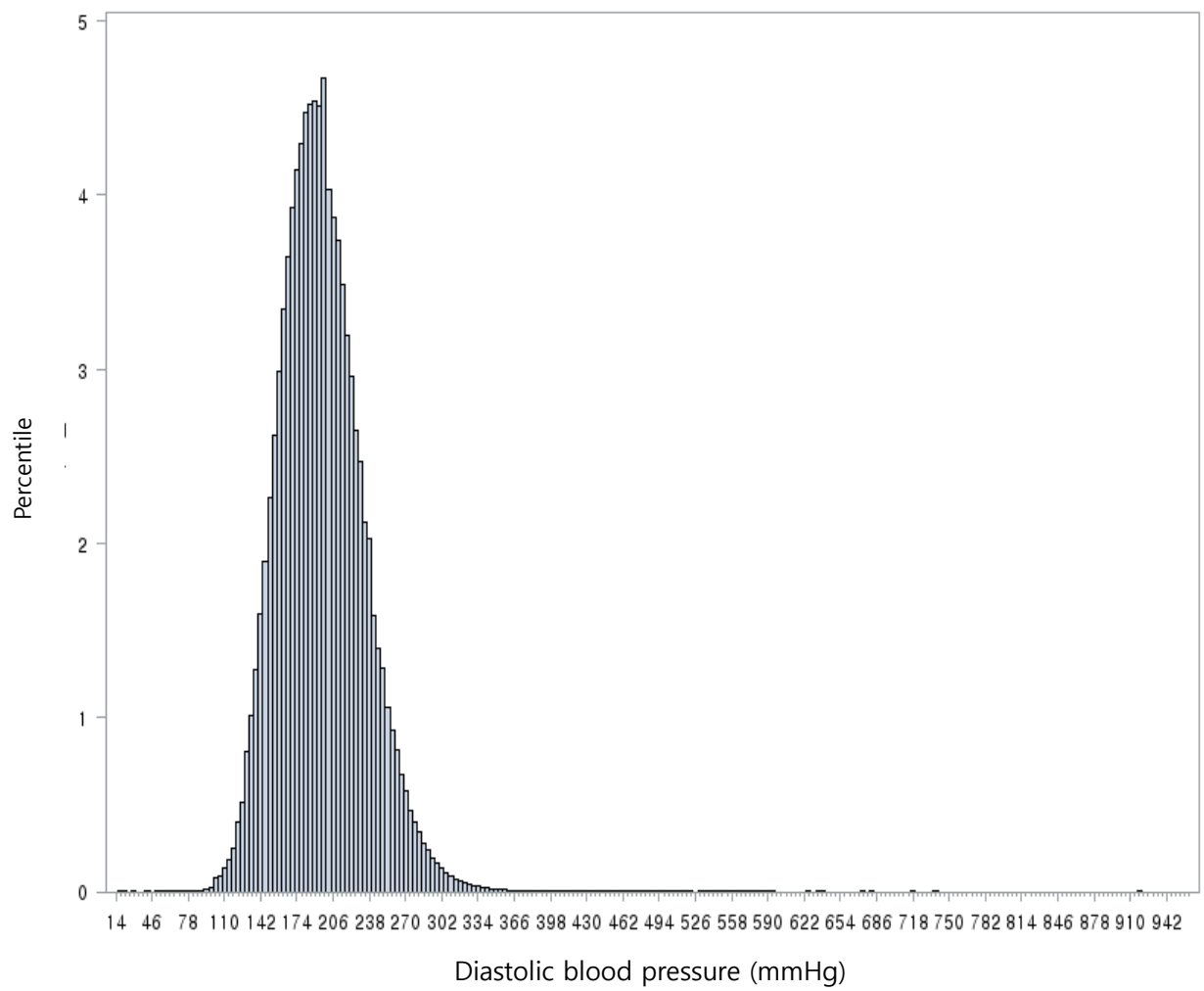

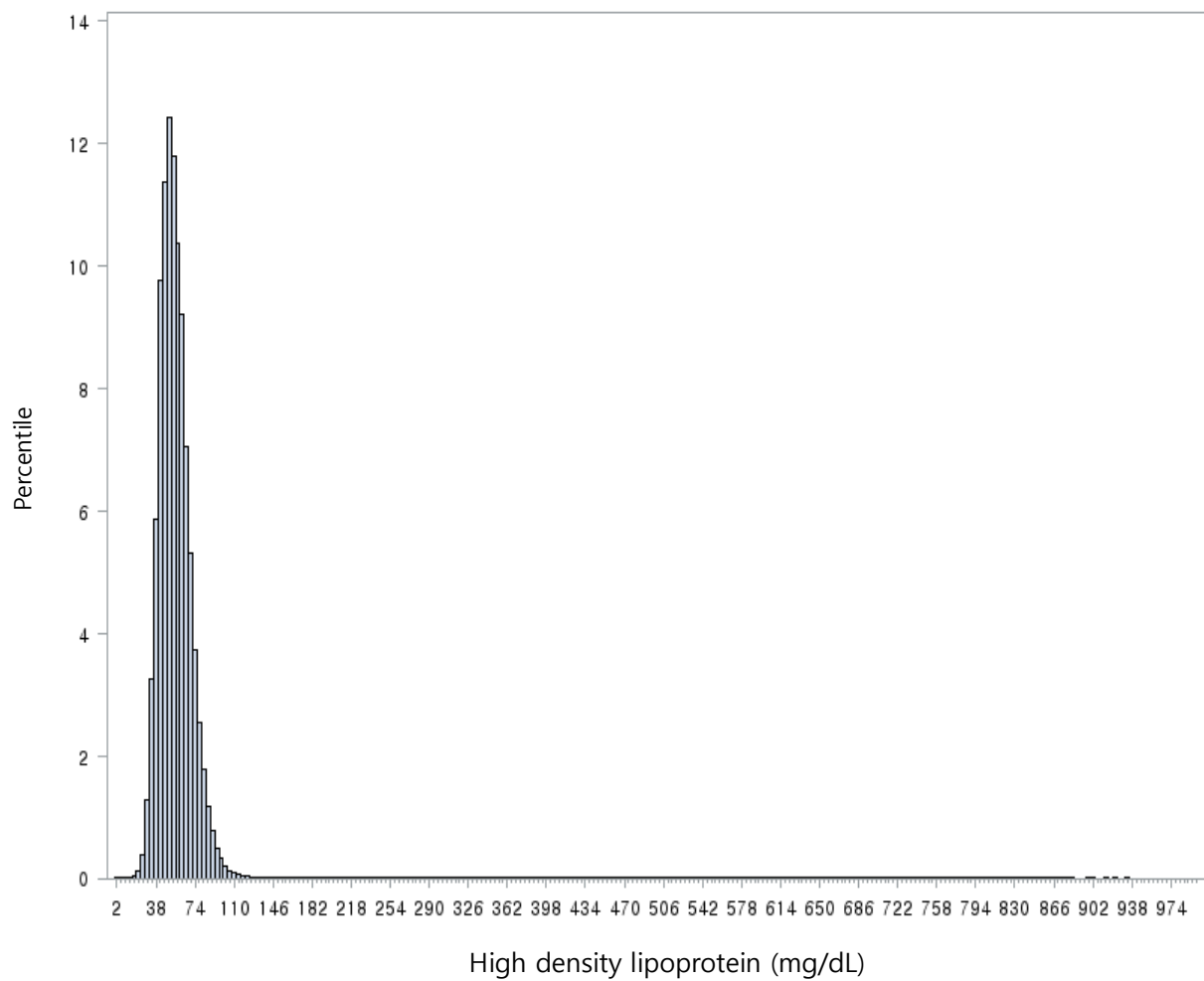

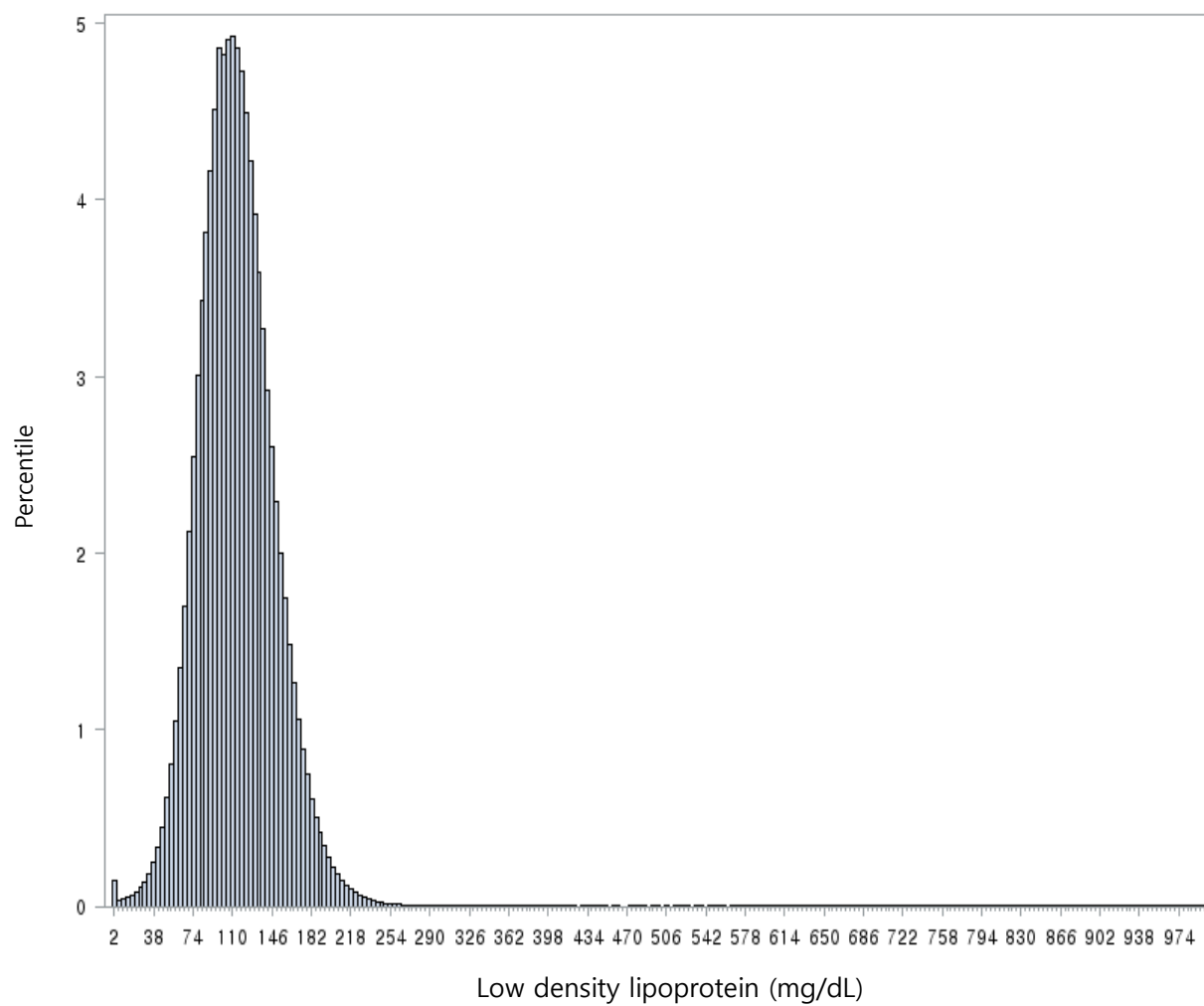

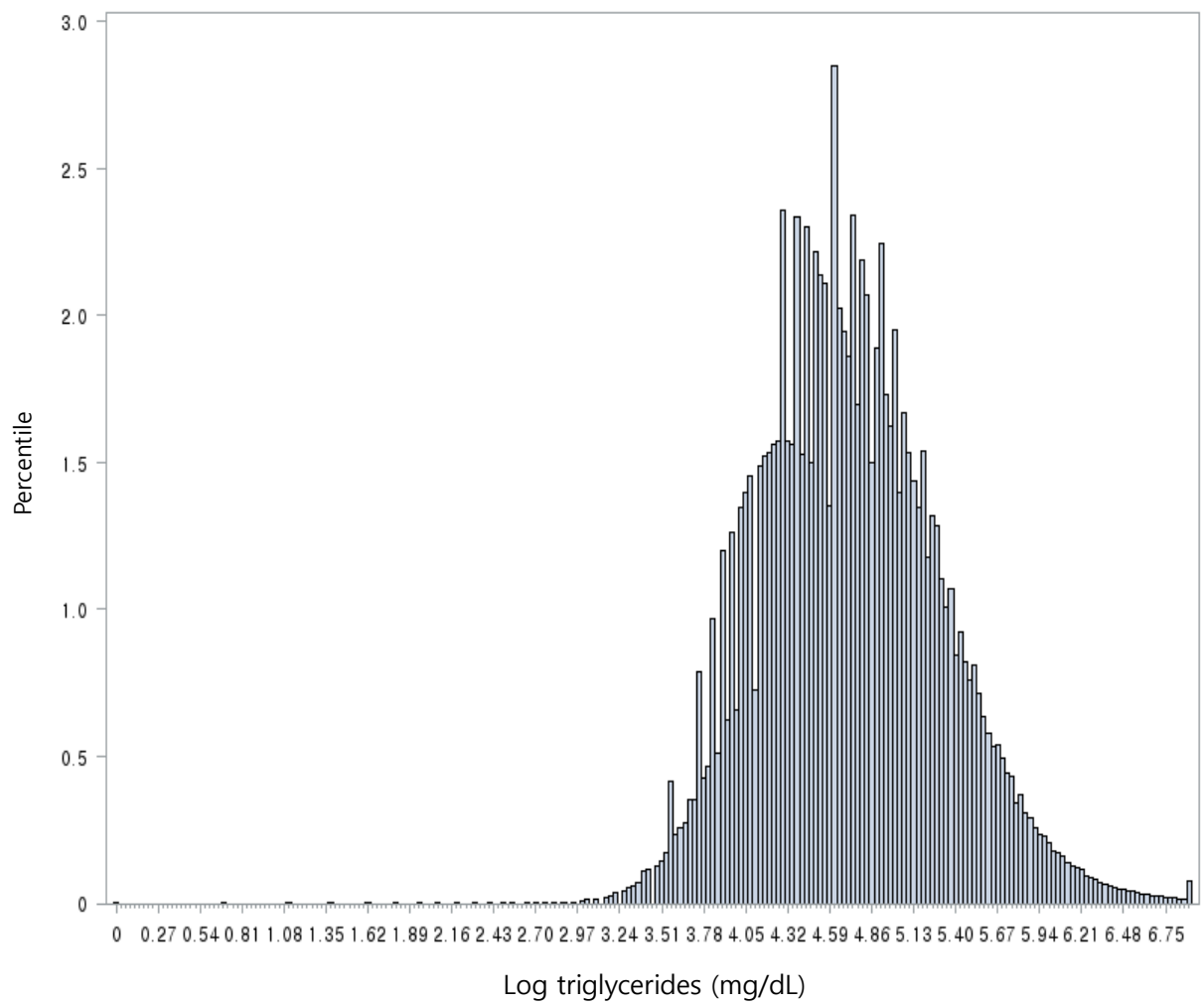

Supplement: Supplementary file 2 — Supplementary Information 2. [file 41598_2021_2517_MOESM2_ESM.pdf]
